# Malignant transformation of fibrous dysplasia: A case report

**DOI:** 10.3892/ol.2014.2082

**Published:** 2014-04-22

**Authors:** HIROSHI HATANO, TETSURO MORITA, TAKASHI ARIIZUMI, HIROYUKI KAWASHIMA, AKIRA OGOSE

**Affiliations:** 1 Department of Orthopedic Surgery, Niigata Cancer Center Hospital, Niigata 951-8566, Japan; 2 Division of Orthopedic Surgery, Department of Regenerative and Transplant Medicine, Niigata University Graduate School of Medical and Dental Sciences, Niigata 951-8510, Japan

**Keywords:** malignant transformation, fibrous dysplasia, secondary osteosarcoma, mutation

## Abstract

Secondary osteosarcoma from fibrous dysplasia (FD) is very rare. The etiology of FD is linked to activating missense mutations of the guanine nucleotide-binding protein α-subunit (*GNAS*) gene, which encodes the stimulatory α subunit of the G protein (G_s_α) and is located at chromosome 20q13. These mutations are central to the pathogenesis of FD; however, it is not known whether G_s_α mutations are retained following malignant transformation in FD. In addition, to the best of our knowledge, no studies have been performed on chromosomal analysis of secondary osteosarcoma from FD. The present study presents a case of secondary osteosarcoma arising from polyostotic FD in a 72-year-old male. Chromosomal analysis showed 44, X, -Y, add(4)(p11), add(5)(p15), der(11)add(11)(p15)t(1;11)(q21;q23), add(12)(q11), -13, der(22)t(12;22)(q11;p12). Reverse transcription-polymerase chain reaction (RT-PCR) analysis demonstrated the presence of a G_s_α mutation in both the primary tumor cells and secondary osteosarcoma cells. There was no alteration in this mutation in the region of malignant transformation, which suggests that this mutation may be a useful clinical marker for distinguishing *de novo* osteosarcoma (primary osteosarcoma) from secondary osteosarcoma arising from FD.

## Introduction

Malignant transformation of fibrous dysplasia (FD) is rare (1–4). It can occur in monostotic and polyostotic FD, with a frequency of <1% among all FD (2). The most common type of malignant tumor arising from FD is osteosarcoma (~70%), followed by fibrosarcoma (~20%), and chondrosarcoma (~10%), with malignant fibrous histiocytoma (~4%) occurring less commonly (2).

Activating missense mutations in the guanine nucleotide-binding protein α-subunit (*GNAS*) gene, which encodes the stimulatory α subunit of the G-protein (G_s_α), resulting in a change at the Arg 201 codon from arginine to cysteine (Arg-to-Cys, R201C) or arginine to histidine (Arg-to-His, R201H) have been identified in both the monostotic and polyostotic forms of FD, as well as in McCune-Albright syndrome (5–7). These mutations are central to the pathogenesis of FD; however, it remains unknown whether the G_s_α mutations are retained following malignant transformation of FD. In addition, to the best of our knowledge, no studies have been performed on chromosomal alterations that occur in FD with malignant transformation. The present study reveals the chromosomal analysis, as well as the status of the G_s_α mutations, of a patient with an osteosarcoma arising in polyostotic FD. Patient provided written informed consent.

## Case report

### Case summary

**Figure 1:**
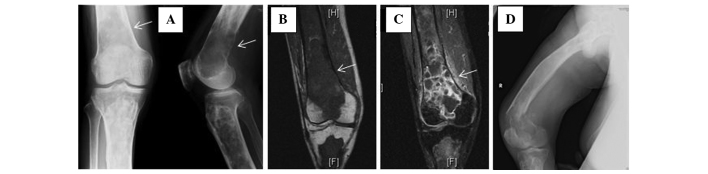
(A) Roentgenograms showing typical fibrous dysplasia involving the tibia and an ill-defined osteolytic lesion (arrow) in the right distal femur. (B) T1-weighted coronal-plane magnetic resonance imaging of the lesion of secondary osteosarcoma (arrow). (C) The lesion was intensively enhanced by injection of gadopentetate dimeglumine (arrow). (D) A pathological fracture through the lesion of the distal femur.

A 72-year-old male presented to the Ojiya General Hospital (Ojiya, Japan) with right knee pain of 3 months in duration, with no history of previous trauma. This patient had a history of colon cancer that had been resected 2 years prior to presentation. There was no recurrence or metastasis. Initial workup roentgenograms, which were performed at the Nagaoka Red Cross Hospital (Nagaoka, Japan) showed a ground glass appearance and a well-defined lucency with sclerotic margins in the right femur and tibia. Although these features are consistent with polyostotic FD, an ill-defined osteolytic lesion, 4×3 cm in size, was superimposed on the changes of FD in the inferior part of the femur (Fig. 1A). Magnetic resonance imaging (MRI) showed the extraosseous extent of the tumor from the lesion in the distal part of the femur (Fig. 1B). The lesion was intensively enhanced by injection of gadopentetate dimeglumine (Fig. 1C), which is suggestive of malignant transformation of FD. The patient did not show cutaneous pigmentation, endocrine disturbances, or soft tissue lesions, as can be seen in McCune-Albright and Mazabraud’s syndromes. No other members of the family had a history of bone tumor. Blood chemistry data showed that the alkaline phosphatase and C-reactive protein levels were elevated to 960 IU/l (normal level, 120–325 IU/l) and 7.39 mg/dl (normal level, <0.3 mg/dl), respectively. Other values, including those of serum calcium (9.4 mg/dl; normal level, 8.7–11.0 mg/dl), phosphorus (4.2 mg/dl; normal level, 2.6–4.4mg/dl), aspartate aminotransferase (24 U/l; normal level, 12–34 U/l), alanine aminotransferase (12 U/l; normal level, 7–36 U/l) and total bilirubin (0.7 mg/dl; normal level, 0.2–1.2 mg/dl), were within acceptable limits. Open biopsy revealed an osteosarcoma with an adjacent area of FD. The patient was referred to the Niigata Cancer Center Hospital (Niigata, Japan) for further treatment of this lesion. Since a pathological fracture through the lesion of the distal femur (see Fig. 1D) had become evident after admission to our hospital, the patient underwent thigh amputation.

### Pathological analysis

**Figure 2:**
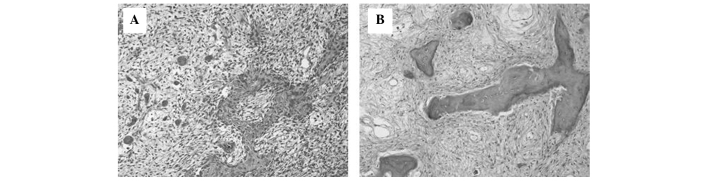
(A) Hypercellular areas show atypical, plump, hyperchromatic stromal cells producing various forms of osteoid. (B) Pre-existing fibrous dysplasia showing bizzarely contoured dysplastic lamellar bone with no osteoblastic rimming.

Gross specimen analysis showed that in the distal femur, the tumor had destroyed part of the cortex and had extended to the surrounding soft tissue. Microscopic examination of this area showed highly pleomorphic, spindle-shaped tumor cells, producing various forms of osteoid (Fig. 2A). The tumor was densely cellular with a high mitotic rate, including atypical figures. The histological features of this area confirmed the diagnosis of osteosarcoma. The adjacent intramedullary part of the tumor in the femur and the tibia showed a solid yellow-white appearance. Microscopic examination of these lesions showed features consistent with FD; small trabeculae of woven bone of various sizes and shapes, scattered within a fibrous tissue without evidence of osteoblastic activity (Fig. 2B). Thus, the case was diagnosed as secondary osteosarcoma arising in pre-existing FD.

### Chromosomal analysis and reverse transcription-polymerase chain reaction (RT-PCR)

**Figure 3:**
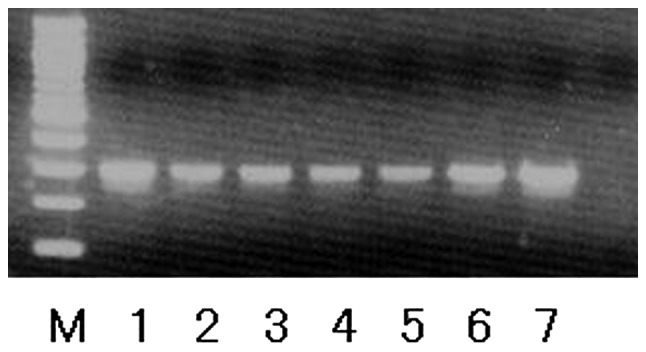
Reverse transcription-polymerase chain reaction analysis for G_s_α mutations in tissue samples. All the samples examined exhibited an activating arginine to histidine point mutation in G_s_α at Arg 201 (R201H). M, 100-bp ladder; 1, positive control; 2 and 3, secondary osteosarcoma; 4, and 5, FD in the femur; 6 and 7, FD in the tibia. FD, fibrous dysplasia.

Chromosomal analysis by G-banded karyotyping showed 44,X,-Y, add(4)(p11), add(5)(p15), der(11)add(11)(p15)t(1;11)(q21;q23),add(12)(q11), -13, der(22)t(12;22)(q11;p12) in nine out of 10 metaphases from the osteosarcoma lesion. RT-PCR analysis for G_s_α mutations, which was performed as previously described (8), demonstrated the presence of a G_s_α mutation at the Arg 201 codon in both the primary tumor cells and secondary osteosarcoma cells (Fig. 3).

### Follow-up

In terms of systemic treatment, adjuvant chemotherapy was not administrated to the patient due to advanced age. At 4 years of clinical follow-up, the patient was well without local recurrence or metastatic disease.

## Discussion

FD is a common benign fibro-osseous lesion; it occurs in 5–7% of all benign bone tumors (9,10). The etiology of FD is linked to activating missense mutations in the *GNAS* gene, which encodes G_s_α and is located at 20q13 (5). G_s_α mutations have been found in tumors from both the monostotic and polyostotic form of FD, as well as in the McCune-Albright syndrome, a disorder that combines polyostotic FD, skin pigmentation and one or several endocrinopathies (9,10). Among fibro-osseous lesions of bone, G_s_α mutations are specific to FD (6–8).

Malignant transformation of FD is very rare (1–4). Thus, the status of G_s_α mutations in osteosarcoma arising from FD has not been reported in the English literature. In the current case, the same G_s_α mutation was detected in both the region of FD and the region of malignant transformation. In addition, chromosomal analysis of the osteosarcoma cells did not show any alterations in chromosome 20, which harbors the *GNAS* gene. From the current study, it is not clear whether the G_s_α mutation itself was directly responsible for the pathogenesis of the malignant transformation of FD. However, the fact that the Gsα mutation did not change through the process of malignant transformation leads us to believe that this mutation has the potential to at least be a clinical marker for distinguishing *de novo* osteosarcoma (primary osteosarcoma) from secondary osteosarcoma arising from FD.

Tumorigenesis in osteosarcoma may involve a complex interplay of chromosomal alternations, with loss of tumor suppressor genes, altered expression of oncogenes and increased levels of certain growth factors (11–13). Although no characteristic chromosome translocations have been identified in osteosarcomas, several chromosomal regions appear to be altered non-randomly (14,15). Bridge *et al* examined 111 chromosomally abnormal osteosarcoma specimens and found that chromosomal regions 1p11–13, 1q10–12, 1q21–22, 11p15, 12p13, 17p12–13, 19q13 and 22q11–13 were most frequently rearranged, and that the most common numerical abnormalities were +1, −9, −10, −13, and −17 (14). Among these, the most thoroughly investigated deletion hotspots are those at 13q14 and 17p13, which correspond with the RB1 and TP53 tumor suppressor genes, respectively. Mutations in TP53 have been shown to result in impaired DNA repair mechanisms and disrupted antiangiogenesis activity (12,15). The RB1 gene is critical to cell-cycle control, and inherited mutations in the RB1 gene cause retinoblastoma syndrome, a condition that predisposes a patient to multiple malignancies (12,15). Mutations or dysfunction in both the TP53 and RB1 genes have also been shown to be involved in osteosarcoma pathogenesis (11,12,15).

Of the abovementioned common chromosomal alterations, loss of chromosome 13 and rearranged chromosomal regions 1q21–22 and 11p15 were found in the current case. The loss of chromosome 13 in this case would have resulted in the inactivation of RB1, which correlated with the malignant transformation of FD. Although the definitive roles of the other chromosomal alterations involved are not known, given the fact that the majority of common chromosomal abnormalities in primary osteosarcoma were also found in secondary osteosarcoma, it seems reasonable to assume that these chromosomal abnormalities also play important roles in tumorigenesis in osteosarcoma.

In summary, the present study reports the presence of a G_s_α mutation and chromosomal alterations in secondary osteosarcoma arising from polyostotic FD. Further investigation is required to elucidate the mechanism and impact of these alterations in the malignant transformation of FD.
